# Simultaneous Occurrence of Two Ectopic Extramammary Paget Disease Lesions: A Case Report

**DOI:** 10.7759/cureus.67589

**Published:** 2024-08-23

**Authors:** Kenji Yorita, Norihiro Hokimoto, Keiko Sakamoto, Satoka Yoshii, Keisuke Kashiwagi

**Affiliations:** 1 Diagnostic Pathology, Japanese Red Cross Kochi Hospital, Kochi, JPN; 2 Surgery, Japanese Red Cross Kochi Hospital, Kochi, JPN; 3 Plastic Surgery, Japanese Red Cross Kochi Hospital, Kochi, JPN

**Keywords:** pathology, surgery, ectopia, extramammary paget disease, skin

## Abstract

Paget disease of the breast is a malignant tumor that occurs primarily on the skin of the nipple, whereas extramammary Paget disease occurs on the skin in regions other than the breast, such as the pubis, perianal area, and axilla. Paget disease can also arise outside these areas; this is referred to as ectopic extramammary Paget disease. In this study, we present a case of a Japanese woman in her 70s who experienced simultaneous occurrence of two ectopic extramammary Paget disease lesions on the skin of the anterior chest and left buttock. Both lesions, exceeding 5 cm in diameter, were surgically excised, and the histopathological examination led to a diagnosis of ectopic extramammary Paget disease without invasion. To the best of our knowledge, this is the third reported case of multiple simultaneous ectopic extramammary Paget disease. Clinically, diagnosis of a patient with ectopic extramammary Paget disease is challenging because of its unusual location; however, the disease may be considered a differential diagnosis for Bowen disease.

## Introduction

Paget disease (PD) is known to occur in two locations: breast and extramammary regions. PD of the breast (mammary PD [MPD]) and extramammary PD (EMPD) are malignant tumors, in which cancer cells grow primarily in the epidermis or the epithelium of the adnexa. Most cases involve intraepithelial carcinomas, although invasive carcinomas can also occur. MPD occurs on the skin of the nipple and areola. EMPD, accounting for 6.5% of all cases of PD [1], occurs in areas with a large number of apocrine sweat glands, most commonly in the pubic region, followed by the perianal region, axilla, and other parts of the body [2, 3]. PD occurring in regions other than the nipple, areola, vulva, perianal area, and axilla has been reported as ectopic EMPD (E-EMPD), with a review of the literature recording only 45 such cases [4]. Of the 45 cases, 64% were Asian, 11% Caucasian, and the rest were unspecified [4]. The incidence of E-EMPD in reports from a single institution is considerably low at 0.78% [5].

In this case report, we present two E-EMPD lesions that were simultaneously found on the skin of the anterior chest and the left buttock. To the best of our knowledge, only two cases of simultaneous multiple E-EMPDs have been reported [6, 7]. Herein, we describe the clinicopathological features of the third case.

## Case presentation

A 75-year-old Japanese female patient was referred to our hospital for the diagnosis and treatment of a brown patch on the left anterior chest. The lesion was noticed 10 years ago, which gradually increased in size and became pruritic. The patient had a history of hypertension but no familial history of breast or ovarian cancer.

**Figure 1:**
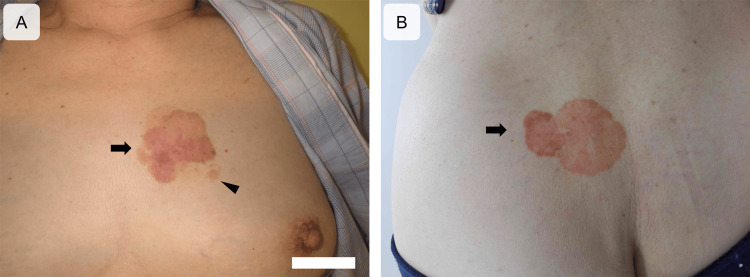
Gross images of ectopic extramammary Paget disease lesions on the left anterior chest (upper inner quadrant of the left breast) and the left buttock Two reddish-brownish skin lesions are observed on the left side of the anterior chest (A, arrow) and on the upper medial side of the left buttock (B, arrow). The anterior chest skin lesion is located in the upper inner quadrant of the left breast (A). The border of these lesions is clear; however, a discontinuous area is observed at the edge of the anterior chest skin lesion (A, arrowhead). These skin lesions are at a distance away from the left nipple, left areola, and perianal area.

The vital signs of the patient were unremarkable. A skin lesion was grossly observed on the left anterior chest in the upper inner quadrant of the left breast, which appeared as a reddish-brownish plaque measuring 5.7 × 5.0 cm (Figure 1A). The skin lesion was located away from the nipple and areola of the left breast, and Bowen disease was clinically suspected.

**Figure 2:**
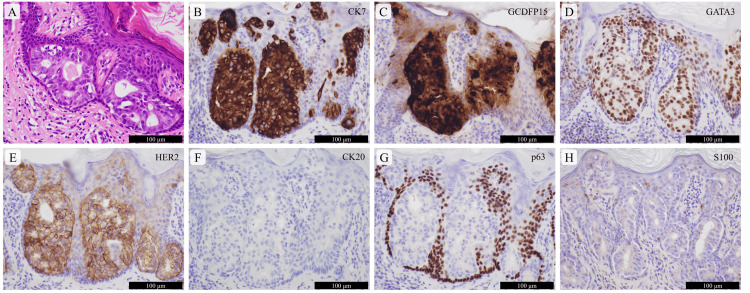
Pathological images of the ectopic extramammary Paget disease lesions on the left anterior chest (upper inner quadrant of the left breast) and the left buttock Atypical cuboidal cells forming tubules are observed in the epidermis (A: hematoxylin and eosin-stained specimen). Immunohistochemically, these tumor cells are diffusely positive for cytokeratin 7 (CK7) (B), gross cystic disease fluid protein 15 (GCDFP15) (C), GATA-binding protein 3 (GATA3) (D), and human epidermal growth factor receptor 2 (HER2) (E) and negative for CK20 (F), p63 (G), and S100 (H). The scale bars are shown in the figure.

The histopathological analysis of the skin biopsy showed atypical cuboidal cells with enlarged nuclei and nucleoli proliferating mainly in a tubular fashion within the epidermis and epithelium of the adnexa. The tumor cells were immunohistochemically positive for cytokeratin 7 (CK7), gross cystic disease fluid protein 15, GATA-binding protein 3, human epidermal growth factor receptor 2, and androgen receptor and negative for CK20, CK5, p63, S100, and CDX2 (Figure 2).

Therefore, this lesion was histologically diagnosed as PD. No neoplastic lesions were observed in the bilateral breasts including nipples and areoras on various modalities such as ultrasonography, contrast-enhanced computed tomography, and contrast-enhanced magnetic resonance imaging. Contrast-enhanced computed tomography from the chest to the pelvis confirmed no abnormal findings in visceral organs. The skin lesion was surgically resected and pathologically confirmed to be PD without invasion. The tumor area measured 21 cm^2^. The lesion was almost consistent with the grossly identified aspect but showed a discontinuous distribution (corresponding to the arrowhead in Figure 1), where a positive margin of less than 1 mm was found. In combination with the gross findings, the lesion was distant from the nipple and areola and a diagnosis of E-EMPD was made.

**Figure 3:**
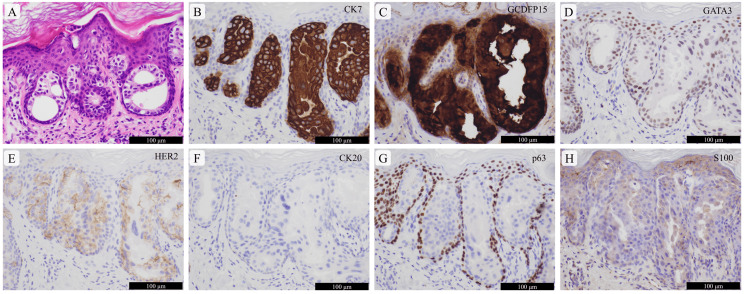
Pathological images of the ectopic extramammary Paget disease of the left buttock Atypical cuboidal cells forming tubules or solid nests are observed in the epidermis (A: hematoxylin and eosin–stained specimen). Immunohistochemically, these tumor cells are diffusely positive for cytokeratin 7 (CK7) (B), gross cystic disease fluid protein 15 (GCDFP15) (C), GATA-binding protein 3 (GATA3) (D), and human epidermal growth factor receptor 2 (HER2) (E) and negative for CK20 (F), p63 (G), and S100 (H). The scale bars are shown in the figure.

Postoperatively, the patient’s daughter noted the presence of a similar skin lesion on the left buttock, which was a reddish-brownish-toned plaque measuring 55 × 45 mm on the upper medial part of the left buttock (Figure 1B). Clinically, Bowen disease was suspected; however, skin biopsy results led to a diagnosis of PD (Figure 3).

A total excision of the skin lesion on the left buttock was performed, and a pathological diagnosis of PD without invasion was confirmed. The tumor area measured 18 cm^2^, and the surgical margin was free of tumor cells. Finally, considering the clinicopathological features, a diagnosis of E-EMPD of the left buttock was made. EMPD was not observed in the vulva, perianal area, and axillae. No skin lesions suggestive of neoplasms, except for surgically resected tumors, were found.

Radiation therapy was administered to the left breast after the surgery, and no recurrence was observed six months postoperatively. The patient provided informed consent for the publication of this report.

## Discussion

MPD is believed to originate from the apocrine sweat glands, which are abundant in the nipples and areola, and from the mammary glands (modified apocrine glands). EMPD is also considered to originate from the apocrine sweat glands, as it occurs in the vulvar, perianal, axillary, and peri-umbilical areas, which are rich in apocrine glands. Modified apocrine glands are also present in the milk line (two longitudinal lines running from the axilla through the nipple to the inguinal region, where adnexal breasts can occur), outer ear (ear canal glands), and eyelids (Moll glands). In a previous review, E-EMPD was defined as PD originating from a region where apocrine glands and modified apocrine glands do not exist [4]. The mechanism underlying the development of E-EMPD is assumed to involve the origin of pluripotent stem cells in the epidermis [4]. In this study, both lesions found on the skin of the anterior chest and left buttock satisfied the definition of E-EMPD.

Differential diseases of E-EMPD include Bowen disease, malignant melanoma, and cutaneous metastasis of salivary gland cancer (especially salivary duct carcinoma) or apocrine carcinoma of the skin. Bowen disease and malignant melanoma could be excluded in our case because the lesions showed no glandular differentiation and returned negative immunohistochemical results for CK5, p63, and S100. Salivary duct carcinoma and apocrine carcinoma show morphological findings and immunostaining results similar to those of this tumor; however, cases of these tumors metastasizing only within the epidermis have not yet been reported. Although intraepithelial extension of urothelial carcinoma or colorectal carcinoma is a differential disease in EMPD, these need not be specifically listed as differential diseases for E-EMPD because E-EMPD is far from the urethral or anal opening, and there are no known cases of urothelial or colorectal cancer metastasizing only within the epidermis. Immunostaining findings also suggested that this tumor was not a metastasis of urothelial carcinoma or colorectal cancer, as urothelial carcinoma is positive for CK7, CK20, and p63, and colorectal cancer is frequently positive for CK20 and CDX2.

E-EMPD has been reported to occur in patients aged between 41 and 90 years, with no particular differences in sex, and tends to be more common in Asians [4]. The most frequent site of occurrence is the trunk, followed by the scalp, extremities, and face [4]. E-EMPD is not clinically diagnosed because it is not found at the usual sites of occurrence, and the clinical presentation can range from eczema to fungal skin infection, contact dermatitis, Bowen disease, spinous cell carcinoma, and malignant melanoma [4]. A biopsy is considered essential for an accurate diagnosis. Using the PubMed database, one case of E-EMPD occurring in the anterior thoracic region, similar to the present case, was identified [8], and only two cases of E-EMPD occurring in the buttocks were reported in a 2010 review [9]. Only two cases of multiple simultaneous E-EMPDs have been reported, similar to our case report [6, 7]: one report showed two simultaneous E-EMPDs on the right lower anterior and left lateral chest regions [6] and the other showed the simultaneous occurrence of a right MPD and 12 E-EMPDs on the anterior chest and back [7]. To our knowledge, this is the first case of multiple simultaneous E-EMPD in the anterior thoracic region and the buttock.

The exact biological malignancy of E-EMPD is unknown owing to the paucity of reports. E-EMPD may have a good prognosis, as there are no reports of single or multiple occurrences of E-EMPD showing invasion [4, 6, 7]. In fact, in the present case report, the pathological diagnosis of the resected specimens showed no invasion in either of the two E-EMPD lesions. However, Weng et al. reported a 36% invasion rate in 44 patients with EMPD with a mean tumor size of 21 cm^2^ [10]. Furthermore, Hatta et al. reported the invasion, metastasis, and mortality rates to be 29%, 17%, and 13%, respectively, in 76 patients with EMPD [11]. More cases of E-EMPD need to be identified to clarify its biological role.

## Conclusions

In this study, we report the case of a woman in her 70s with multiple simultaneous E-EMPD lesions on the skin of the left anterior chest and left buttock. Initially, the two lesions were clinically diagnosed as Bowen disease. Histopathological analysis of the skin biopsy obtained from excision led to the diagnosis of E-EMPD without invasion. To our knowledge, this is the second case of multiple simultaneous E-EMPDs in Japan and the third case worldwide. More cases are desirable for determining the clinicopathological characteristics of this rare disease manifestation.
